# Cell Type-Specific Subcellular Localization of Phospho-TBK1 in Response to Cytoplasmic Viral DNA

**DOI:** 10.1371/journal.pone.0083639

**Published:** 2013-12-09

**Authors:** Takayuki Suzuki, Hiroyuki Oshiumi, Moeko Miyashita, Hussein Hassan Aly, Misako Matsumoto, Tsukasa Seya

**Affiliations:** Department of Microbiology and Immunology, Graduate School of Medicine, Hokkaido University, Sapporo, Hokkaido, Japan; Drexel University College of Medicine, United States of America

## Abstract

Cytoplasmic viral RNA and DNA are recognized by RIG-I-like receptors and DNA sensors that include DAI, IFI16, DDX41, and cGAS. The RNA and DNA sensors evoke innate immune responses through the IPS-1 and STING adaptors. IPS-1 and STING activate TBK1 kinase. TBK1 is phosphorylated in its activation loop, leading to IRF3/7 activation and Type I interferon (IFN) production. IPS-1 and STING localize to the mitochondria and endoplasmic reticulum, respectively, whereas it is unclear where phosphorylated TBK1 is localized in response to cytoplasmic viral DNA. Here, we investigated phospho-TBK1 (p-TBK1) subcellular localization using a p-TBK1-specific antibody. Stimulation with vertebrate DNA by transfection increased p-TBK1 levels. Interestingly, stimulation-induced p-TBK1 exhibited mitochondrial localization in HeLa and HepG2 cells and colocalized with mitochondrial IPS-1 and MFN-1. Hepatitis B virus DNA stimulation or herpes simplex virus type-1 infection also induced p-TBK1 mitochondrial localization in HeLa cells, indicating that cytoplasmic viral DNA induces p-TBK1 mitochondrial localization in HeLa cells. In contrast, p-TBK1 did not show mitochondrial localization in RAW264.7, L929, or T-23 cells, and most of p-TBK1 colocalized with STING in response to cytoplasmic DNA in those mammalian cells, indicating cell type-specific localization of p-TBK1 in response to cytoplasmic viral DNA. A previous knockout study showed that mouse IPS-1 was dispensable for Type I IFN production in response to cytoplasmic DNA. However, we found that knockdown of *IPS-1* markedly reduced p-TBK1 levels in HeLa cells. Taken together, our data elucidated the cell type-specific subcellular localization of p-TBK1 and a cell type-specific role of IPS-1 in TBK1 activation in response to cytoplasmic viral DNA.

## Introduction

RIG-I-like receptors (RLRs) are cytoplasmic viral RNA sensors that play an essential role in Type I interferon (IFN) expression in response to RNA virus infection [1]. RLRs recognize cytoplasmic double-stranded RNA (dsRNA) and the dsRNA analog polyI:C [1]. A recent study reported that RLRs localize on antiviral stress granules in response to cytoplasmic polyI:C or viral infection [2]. IPS-1 (also called MAVS, Cardif, and VISA) is a solo adaptor of RLRs and localizes on the outer-membrane of mitochondria and peroxisomes [3–7]. A recent study reported that a part of IPS-1 localizes on mitochondria-associated membranes (MAMs), which is a distinct membrane compartment that links the endoplasmic reticulum (ER) to the mitochondria [8]. RIG-I is then recruited to MAMs to bind IPS-1 [8]. There are several regulatory proteins on mitochondria such as MFN-1 and MFN-2 [9,10]. Association of RLRs with IPS-1 induces the formation of IPS-1 prion-like aggregates, leading to TBK1 activation [11] and consequent Type I IFN production [12,13]. Toll-like receptor 3 (TLR3) also recognizes viral dsRNA and polyI:C; however, TLR3 localizes to early endosomes or the cell surface and requires the adaptor TICAM-1 to induce Type I IFN expression [14–16]. 

Cytoplasmic DNA sensors, such as DAI, IFI16, DDX41, cGAS, and Mre11, recognizes DNA viruses [17–19]. These DNA sensors recognize not only viral DNA but also cytoplasmic vertebrate or bacterial DNA [20,21]. RLRs are also involved in sensing cytoplasmic DNA [22,23]. Chen and colleagues have shown that DNA viruses can activate RIG-I pathway via RNA polymerase III [24]. Unlike RLRs, the DAI, IFI16, DDX41, and cGAS DNA sensors require the adaptor molecule STING to induce Type I IFN expression [19,25,26]. STING localizes to the ER and requires TBK1 to induce Type I IFN expression [19]. 

The protein kinase TBK1 is essential for Type I IFN expression in response to cytoplasmic DNA [27]. Ser-172 of TBK1 is autophosphorylated in its activation loop, and autophosphorylation is essential for triggering TBK1-dependent signaling [28]. Active TBK1 phosphorylates the transcription factor IRF-3, leading to relocalization of IRF-3 from cytoplasm to nucleus [29]. Recently, we showed that phospho-TBK1 (p-TBK1) localizes on mitochondria in response to cytoplasmic hepatitis C virus RNA [30]; however, it is unclear where TBK1 localizes in response to cytoplasmic viral DNA. Here, we used an anti-p-TBK1 specific antibody to determine the subcellular localization of p-TBK1 in response to cytoplasmic viral DNA. We elucidated the cell type-specific subcellular localization of p-TBK1 in response to cytoplasmic viral DNA. 

## Results

### Localization of p-TBK1 on mitochondria in HeLa cells

**Figure 1:**
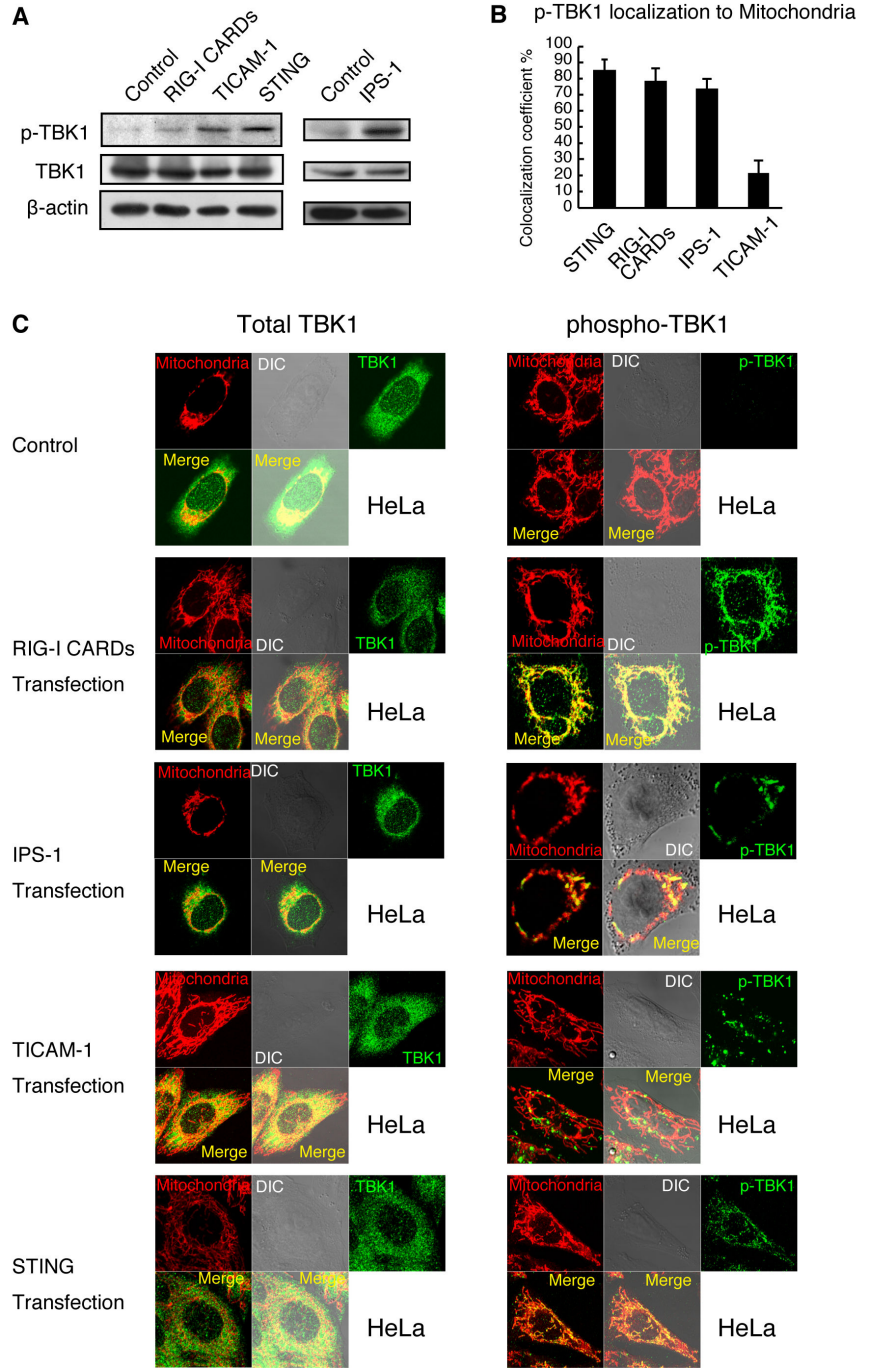
Mitochondrial localization of p-TBK1 in HeLa cells. (A) HeLa cells were transfected with 1.2 μg of empty vector, RIG-I CARDs, TICAM-1, IPS-1, or STING expression vectors in 6-well plate. At 24 h after transfection, cell lysates were prepared and subjected to SDS-PAGE. Proteins were detected by western blotting using anti-TBK1, p-TBK1, and β-actin antibodies. (B and C) HeLa cells were transfected with 0.3 μg of empty vector or RIG-I CARDs, TICAM-1, or STING expression vectors in 24-well plate. At 24 h after transfection, cells were fixed and stained with anti-TBK1 or anti-p-TBK1 antibodies and Mitotracker Red. Colocalization coefficients of p-TBK1 with mitochondria were determined (mean ± sd, n = 3) (C). Unless otherwise indicated, Data are from one representative (n = 3) of at least three independent experiments.

We used anti-TBK1 (total TBK1) and anti-p-TBK1 antibodies to detect total TBK1 and p-TBK1 expression by western blotting and immunofluorescence microscopy analyses. Exogenous expression of RIG-I CARDs, TICAM-1, IPS-1, or STING induces the activation of downstream signaling without stimulation [4,14,26,31]. We found that exogenous expression of RIG-I CARDs, TICAM-1, IPS-1, or STING induced TBK1 phosphorylation, whereas total TBK1 levels were not affected (Figure 1A). We investigated the subcellular localization of p-TBK and total TBK1. Total TBK1 was dispersed through the cytoplasm, whereas p-TBK1 exhibited mitochondrial localization in HeLa cells that expressed RIG-I CARDs, IPS-1, or STING (Figure 1B and 1C). More than 70 % of p-TBK1 induced by RIG-I CARDs, IPS-1, or STING expression showed mitochondrial localization (Figure 1B). In contrast, p-TBK1 did not show mitochondrial localization in HeLa cells that expressed TICAM-1 (Figure 1B and 1C). These data suggested that the activation of RIG-I, IPS-1, or STING signaling, but not TICAM-1 signaling, induced p-TBK1 mitochondrial localization in HeLa cells.

**Figure 2:**
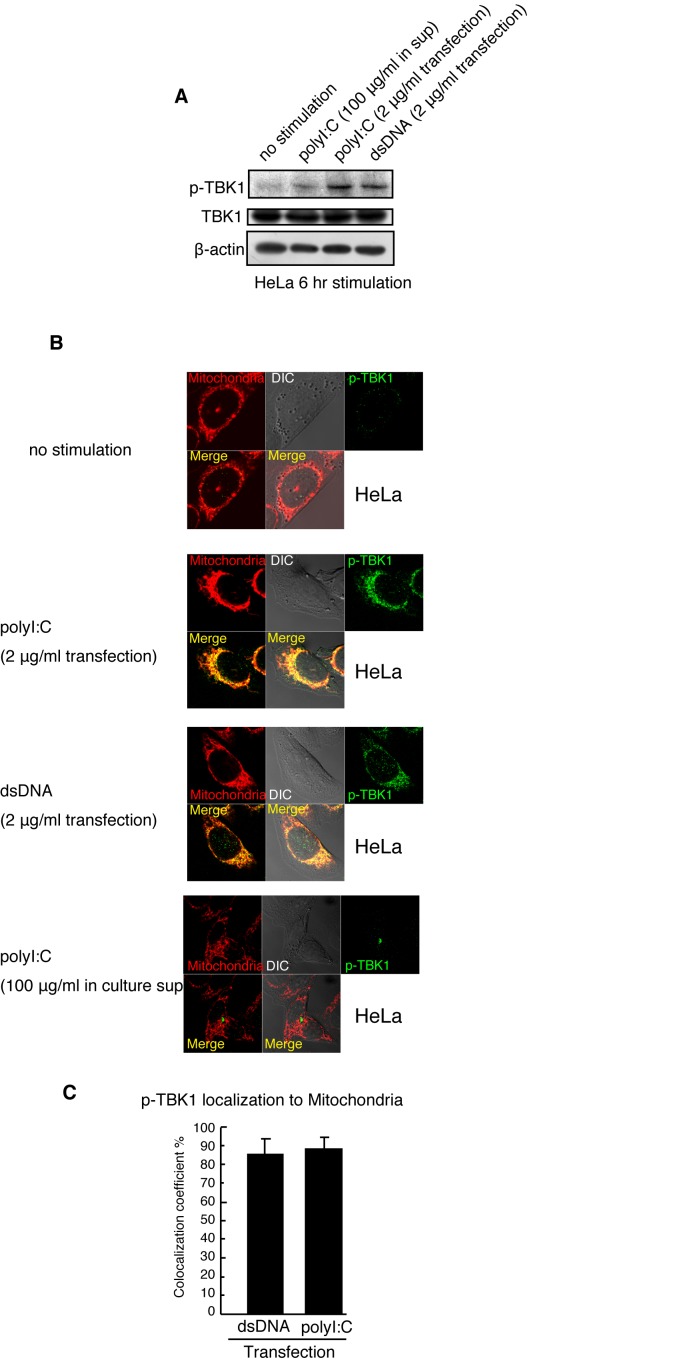
Mitochondrial localization of p-TBK1 in response to cytoplasmic nucleic acids in HeLa cells. (A) HeLa cells were stimulated with 100 μg/ml of polyI:C (no transfection), 2 μg/ml of polyI:C (by transfection) or 2 μg/ml of salmon sperm dsDNA (by transfection) in 6-well plate. At 6 h after stimulation, cell lysates were prepared and subjected to SDS-PAGE. Proteins were detected by western blotting using anti-TBK1, p-TBK1, and β-actin antibodies. (B) HeLa cells were stimulated with 100 μg/ml of polyI:C (no transfection), 2 μg/ml of polyI:C (by transfection), or 2 μg/ml of salmon sperm dsDNA (by transfection) in 24-well plate. At 6 h after stimulation, cells were fixed and stained with anti p-TBK1 antibody and Mitotracker Red. (C) Colocalization coefficients of p-TBK1 with mitochondria were determined (mean ±sd, n = 3).

Next, we examined the localization of p-TBK1 in HeLa cells after polyI:C or vertebrate dsDNA (salmon sperm DNA) stimulation. Previous studies reported that cytoplasmic vertebrate DNA induces Type I IFN expression [21,32]. When HeLa cells were stimulated with transfected polyI:C or dsDNA for 6 h, p-TBK1 levels increased (Figure 2A), and more than 80 % of p-TBK1 showed mitochondrial localization (Figure 2B and 2C). In contrast, when HeLa cells were stimulated with polyI:C without transfection to activate the TLR3 pathway, most of p-TBK1 did not localize on mitochondria (Figure 2B). These data indicated that p-TBK1 localized on mitochondria in response to cytoplasmic polyI:C or dsDNA but not to extracellular polyI:C. 

**Figure 3:**
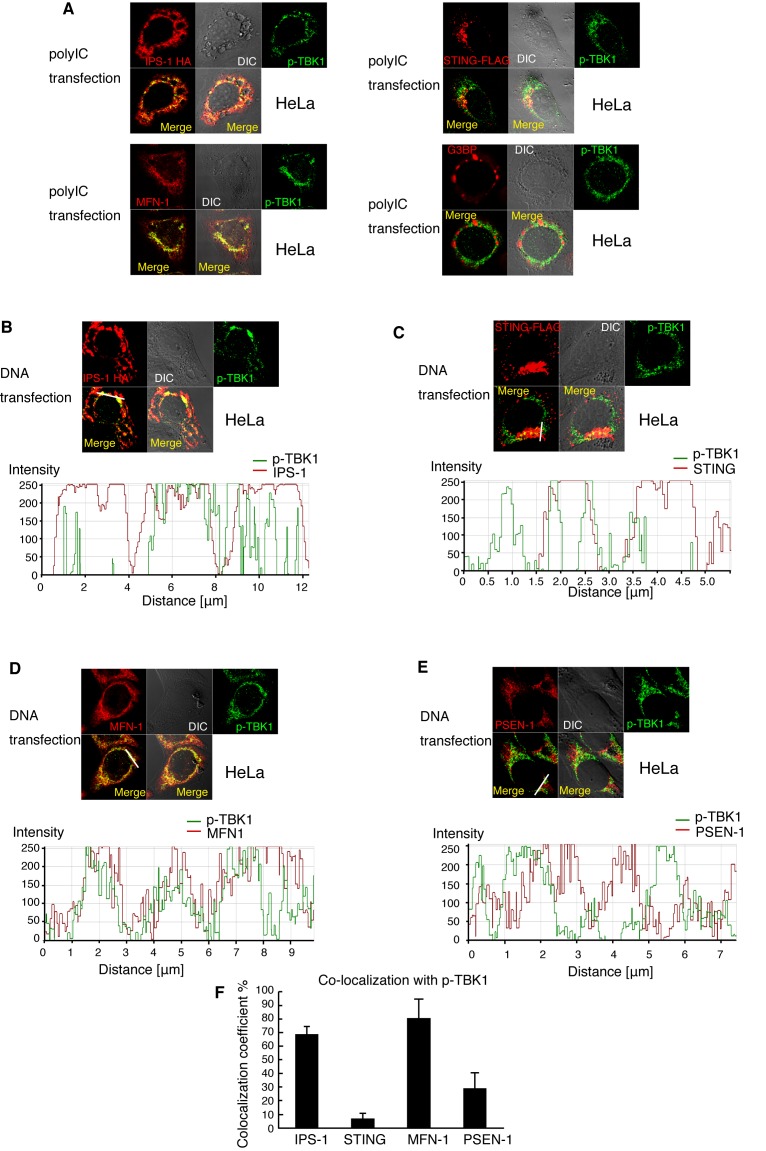
Co-localization of p-TBK1 with IPS-1 in HeLa cells. (A) HeLa cells were transfected with 1 μg of polyI:C in 24-well plate. At 6 h after transfection, cells were fixed and stained with anti-HA, p-TBK1, FLAG, MFN-1, and/or G3BP antibodies. To observe IPS-1 and STING localization, HeLa cells were transfected with HA-tagged IPS-1 or FLAG-tagged STING expression vectors 24 h before stimulation. (B and C) HeLa cells were transfected with 0.3 μg of HA-tagged IPS-1 (B) or FLAG-tagged STING (C) expression vectors. At 24 h after transfection, HeLa cells were stimulated with 1 μg of salmon sperm dsDNA by transfection in 24-well plate. At 6 h after stimulation, cells were fixed and stained with anti-p-TBK1 and anti-HA or FLAG antibodies. Histograms display the measured fluorescence intensity along the white line in the merged panels. (D and E) HeLa cells were stimulated with salmon sperm dsDNA by transfection. At 6 h after stimulation, cells were fixed and stained with anti-p-TBK1 and MFN-1 (D) or PSEN-1 antibodies (E). Histograms display the measured fluorescence intensity along the white line in the merged panels. (F) Colocalization of coefficients of p-TBK1 with IPS-1, STING, MFN-1 or PSEN-1 are shown (mean ± sd, n = 3).

Next, we compared the subcellular localization of p-TBK1 to other proteins after polyI:C transfection. We found that p-TBK1 colocalized with a mitochondrial protein MFN-1 (Figure 3A). In contrast, p-TBK1 barely colocalized with the stress granule marker G3BP (Figure 3A). To compare the p-TBK1 localization with IPS-1 and STING localizations, HA-tagged IPS-1 or FLAG-tagged STING were transfected into HeLa cells. At 24 h after transfection, cells were stimulated by mock or polyI:C transfection for 6 h. Although either IPS-1 or STING expression induced p-TBK1 staining without stimulation (Figure S1A and S1B), most p-TBK1 colocalized with HA-tagged IPS-1 but poorly colocalized with FLAG-tagged STING in both stimulated and mock-stimulated cells (Figure 3A and Figure S1). These data are consistent with previous observations that IPS-1, but not STING, is essential for Type I IFN production in response to polyI:C [6,25]. 

Next, we stimulated HeLa cells by dsDNA transfection. Interestingly, p-TBK1 colocalized with exogenously expressed HA-tagged IPS-1 (Figure 3B) in dsDNA stimulated HeLa cells, although IPS-1 is known to be dispensable for type I IFN production in response to DNA stimulation [33]. We found that p-TBK1 induced by DNA stimulation colocalized with a mitochondria marker MFN-1, and partially colocalized with a MAMs marker Presenilin-1 (PSEN-1) and exogenously expressed FLAG-tagged STING (Figure 3C-3E). Statistical analysis suggested that more than 60 % of p-TBK1 colocalized with HA-tagged IPS-1 and MFN-1, whereas less than 10 % of p-TBK1 colocalized with FLAG-tagged STING (Figure 3F). Approximately 30 % of p-TBK1 colocalized with PSEN-1 (Figure 3F). Taken together, these data suggested that most mitochondrial p-TBK1 induced by DNA transfection colocalized with IPS-1 and MFN-1 in HeLa cells. Because STING but not IPS-1 is essential for Type I IFN expression in response to cytoplasmic DNA [25,33], there appears to be an apparent contradiction between our subcellular localization and previous genetic data. Thus, we further focused on p-TBK1 localization induced by cytoplasmic DNA to dissect these apparently contradictory results.

### Cell Type-Specific Localization of p-TBK1 in Response to Cytoplasmic DNA

**Figure 4:**
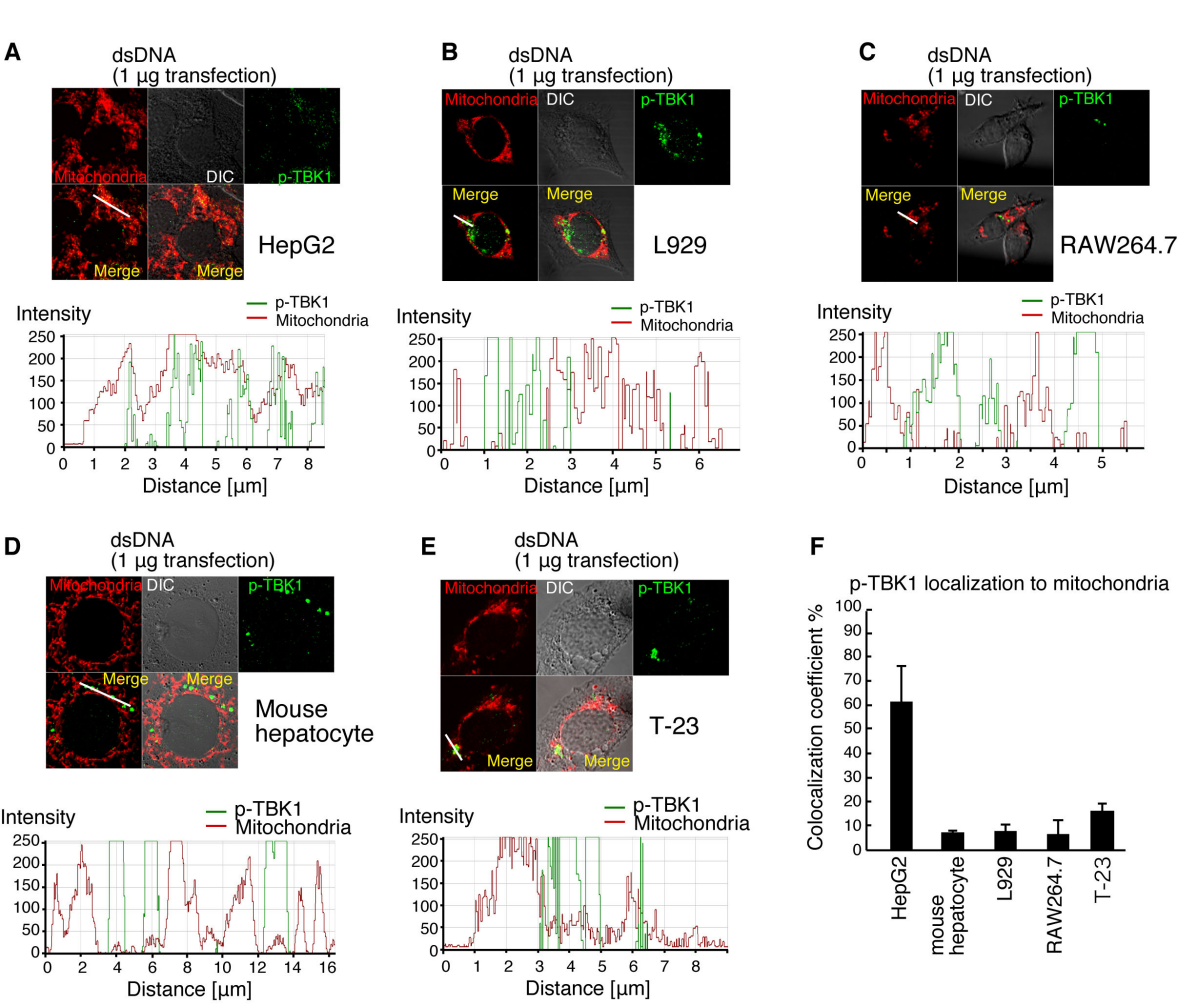
Cell type-specific localization of p-TBK1 in response to cytoplasmic dsDNA. (A-E) HepG2 (A), L929 (B), RAW264.7 (C), mouse hepatocyte (D), and T-23 (E) cells were stimulated with salmon sperm dsDNA by transfection. At 6 h after stimulation, cells were fixed and stained with anti-p-TBK1 antibody and Mitotracker Red. Histograms display the measured fluorescence intensity along the white line in the merged panels. (F) Colocalization coefficients of p-TBK1 with mitochondria are shown (mean ± sd, n = 3).

**Figure 5:**
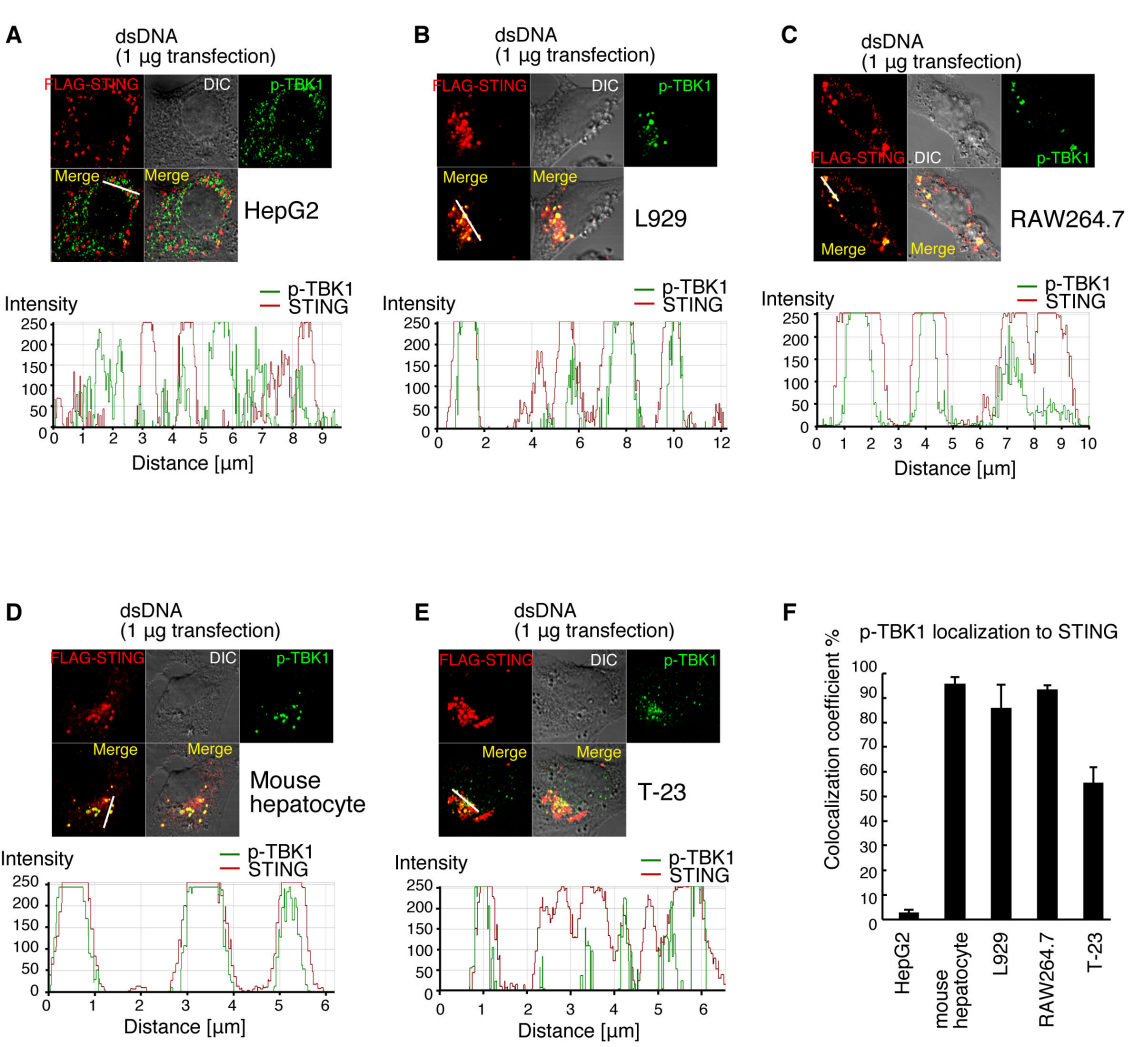
p-TBK1 colocalization with STING in mammalian cells in response to DNA. (A-E) 0.5 μg of FLAG-tagged STING expression vector was transfected into HepG2 (A), L929 (B), RAW264.7 (C), mouse hepatocytes (D) or T-23 (E) cells. At 24 h after transfection, cells were stimulated with salmon sperm dsDNA. At 6 h after transfection, cells were fixed and stained with anti-FLAG and p-TBK1 antibodies. Histograms display the measured fluorescence intensity along the white line in the merged panels. (F) Colocalization coefficients of p-TBK1 with STING are shown (mean ± sd, n = 3).

We investigated whether p-TBK1 induced by DNA transfection exhibited mitochondrial localization in other cell lines. As seen with HeLa cells, in HepG2 cells, p-TBK1 exhibited mitochondrial localization in response to cytoplasmic DNA (Figure 4A and 4F). In contrast, most p-TBK1 did not exhibit mitochondrial localization in L929, RAW264.7, a mouse hepatocyte cell line [34], or tree shrew fibroblast T-23 cells [35] (Figure 4B-4E). Statistical analysis showed that fewer than 20% of p-TBK1 localized on mitochondria in the mouse hepatocyte cell line, L929, RAW264.7, and tree shrew T-23 cells (Figure 4F). Although p-TBK1 colocalized with exogenously expressed HA-tagged IPS-1 but not FLAG-tagged STING in dsDNA stimulated HeLa cells (Figure 3B and 3C), most p-TBK1 colocalized with exogenously expressed FLAG-tagged STING in dsDNA stimulated L929, RAW264.7, mouse hepatocytes or T-23 cells but not in HepG2 (Figure 5A-5F). These data suggested that p-TBK1 exhibited cell type-specific localization in response to cytoplasmic DNA. 

### p-TBK1 mitochondrial localization in response to cytoplasmic viral DNA in human cell lines

**Figure 6:**
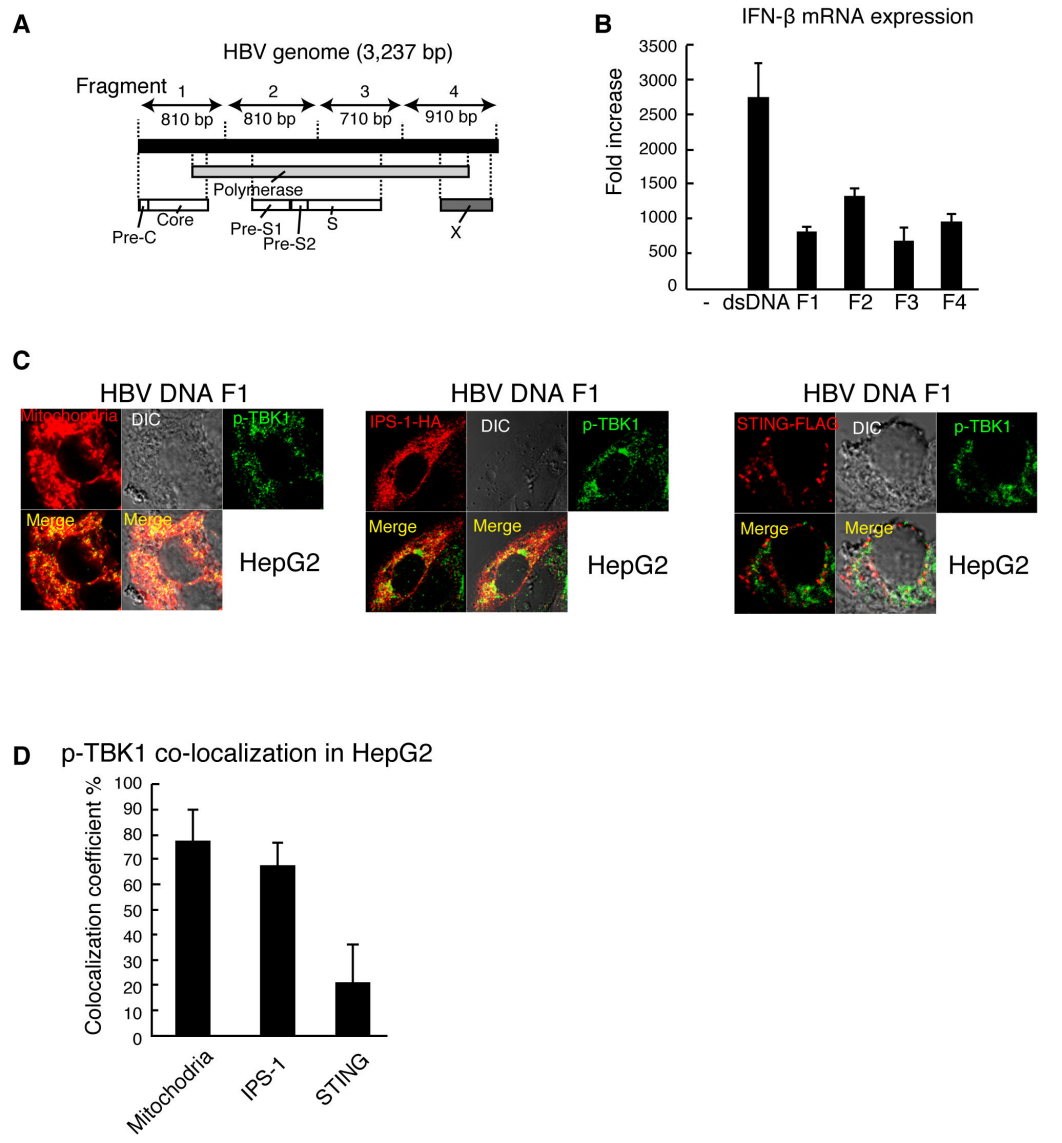
p-TBK1 colocalization with IPS-1 in response to a HBV genomic DNA fragment in HepG2 cells. (A) A schematic diagram of HBV genomic DNA fragments used in panels B-J. (B) RAW264.7 cells were transfected with mock, salmon sperm dsDNA, or HBV dsDNA fragments F1, F2, F3, or F4. At 6 h after transfection, IFN-β mRNA expression was determined by RT-qPCR. (C) HepG2 cells were transfected with 1 μg of HBV fragment 1 in 24-well plate. At 6 h after transfection, cells were fixed and stained with anti-p-TBK1 antibody and Mitotracker Red or anti-HA antibody. To observe IPS-1 and STING localization, 0.5 μg of HA-tagged IPS-1 or FLAG-tagged STING expression vector was transfected into HepG2 cells 24 h before stimulation. (D) Colocalization coefficients of p-TBK1 with mitochondria, IPS-1, or STING (mean ± sd, n = 3).

**Figure 7:**
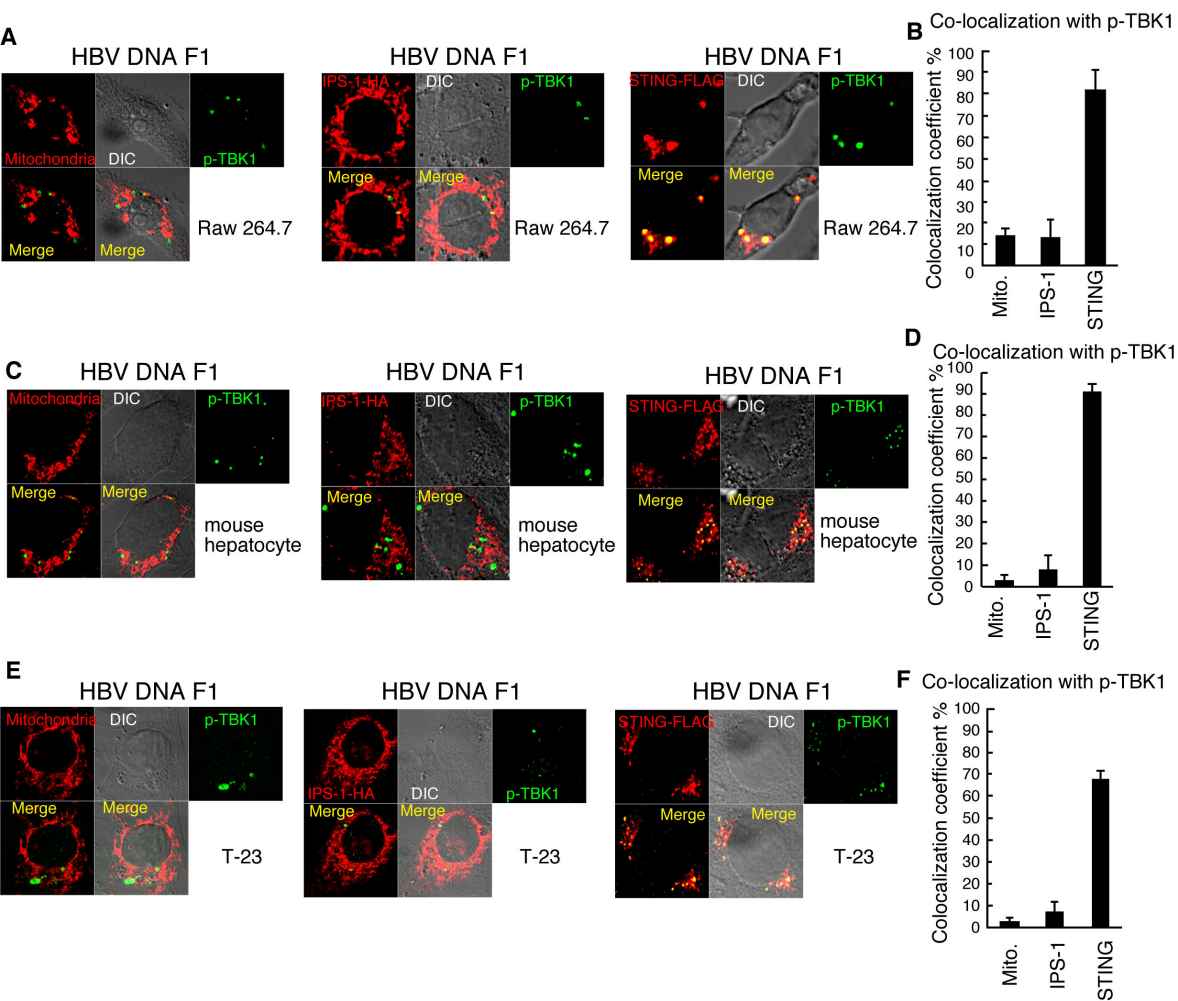
p-TBK1 colocalization with STING in response to a HBV genomic DNA fragment in mammalian cells. RAW264.7 (A and B), mouse hepatocyte (C and D), and T-23 (E and F) cells were transfected with HBV fragment 1. At 6 h after transfection, cells were fixed and stained with anti-p-TBK1 and FLAG antibodies and Mitotracker Red. To observe STING and IPS-1 localization, 0.5 μg of HA-tagged IPS-1 or FLAG-tagged STING expression vectors were transfected into RAW264.7 cells 24 h before stimulation. Colocalization coefficients of p-TBK1 with mitochondria (Mito.), IPS-1, or STING (mean ± sd, n = 3) in RAW264.7 (B), mouse hepatocyte (D), and T-23 (F) cells are shown.

Next, we investigated p-TBK1 localization in response to viral DNA. Hepatitis B virus (HBV) is a DNA virus, and its protein HBx suppresses IFN-β mRNA expression in response to dsDNA but not to dsRNA [36], suggesting that DNA sensing pathway is targeted by HBV. When HBV full-length genomic DNA was transfected into HepG2, type I IFN mRNA expression was hardly induced (Figure S2). To avoid the suppression of innate immune response by HBV proteins transcribed from full-length HBV DNA, we used partial fragments of HBV genomic DNA (F1-F4) (Figure 6A). Stimulation with HBV genomic DNA fragments (F1-F4) efficiently induced *IFN-β* mRNA expression (Figure 6A and 6B). As observed with vertebrate DNA, the HBV genomic DNA fragment F1 induced mitochondrial localization of p-TBK1 (Figure 6C and 6D), and most p-TBK1 colocalized with IPS-1 but not STING in HepG2 cells (Figure 6C and 6D). When RAW264.7, the mouse hepatocyte cell line, or T-23 cells were stimulated with the HBV DNA fragment by transfection, p-TBK1 did not exhibit mitochondrial localization (Figure 7A, 7C, and 7E), and more than 70% of p-TBK1 colocalized with STING (Figure 7B, 7D, and 7F).

**Figure 8:**
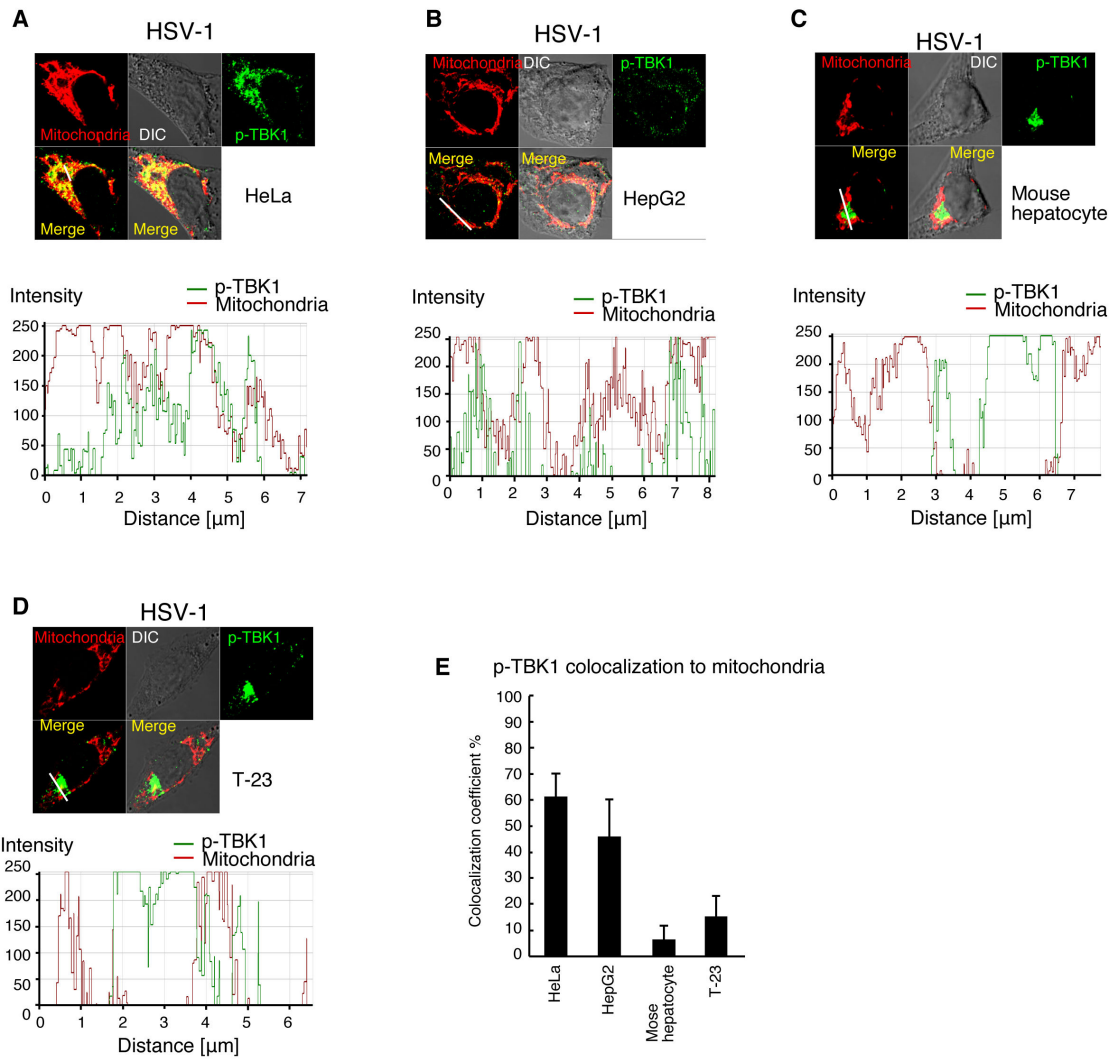
p-TBK1 localization in response to HSV-1 infection. HeLa (A), HepG2 (B), mouse hepatocyte (C), and T-23 (D) cells were infected with HSV-1 for 24 h. Cells were fixed and stained with Mitotracker Red and anti-p-TBK1 antibody. Histograms display the measured fluorescence intensity along the white line in the merged panels. Colocalization coefficients of p-TBK1 to mitochondria are shown (E).

Next, we investigated p-TBK1 localization after a DNA virus herpes simplex virus type-1 (HSV-1) infection in HeLa, HepG2, the mouse hepatocyte cell line, and T-23 cells. In HeLa and HepG2 cells, p-TBK1 localized on mitochondria, whereas, in the mouse hepatocyte cell line and T-23 cells, most p-TBK1 failed to localize on mitochondria (Figure 8A-8D). The statistic analysis indicated that the colocalization coefficient of p-TBK1 to mitochondria of HeLa or HepG2 cells was higher than that of mouse hepatocyte or T-23 cells (Figure 8E). Taken together, these data indicated that p-TBK1 exhibited mitochondrial localization in response to cytoplasmic viral DNA in HeLa and HepG2 cells, but not in RAW264.7, the mouse hepatocyte cell line, or T-23 cells. 

### IPS-1 is essential for TBK1 phosphorylation in HeLa cells in response to cytoplasmic DNA

**Figure 9:**
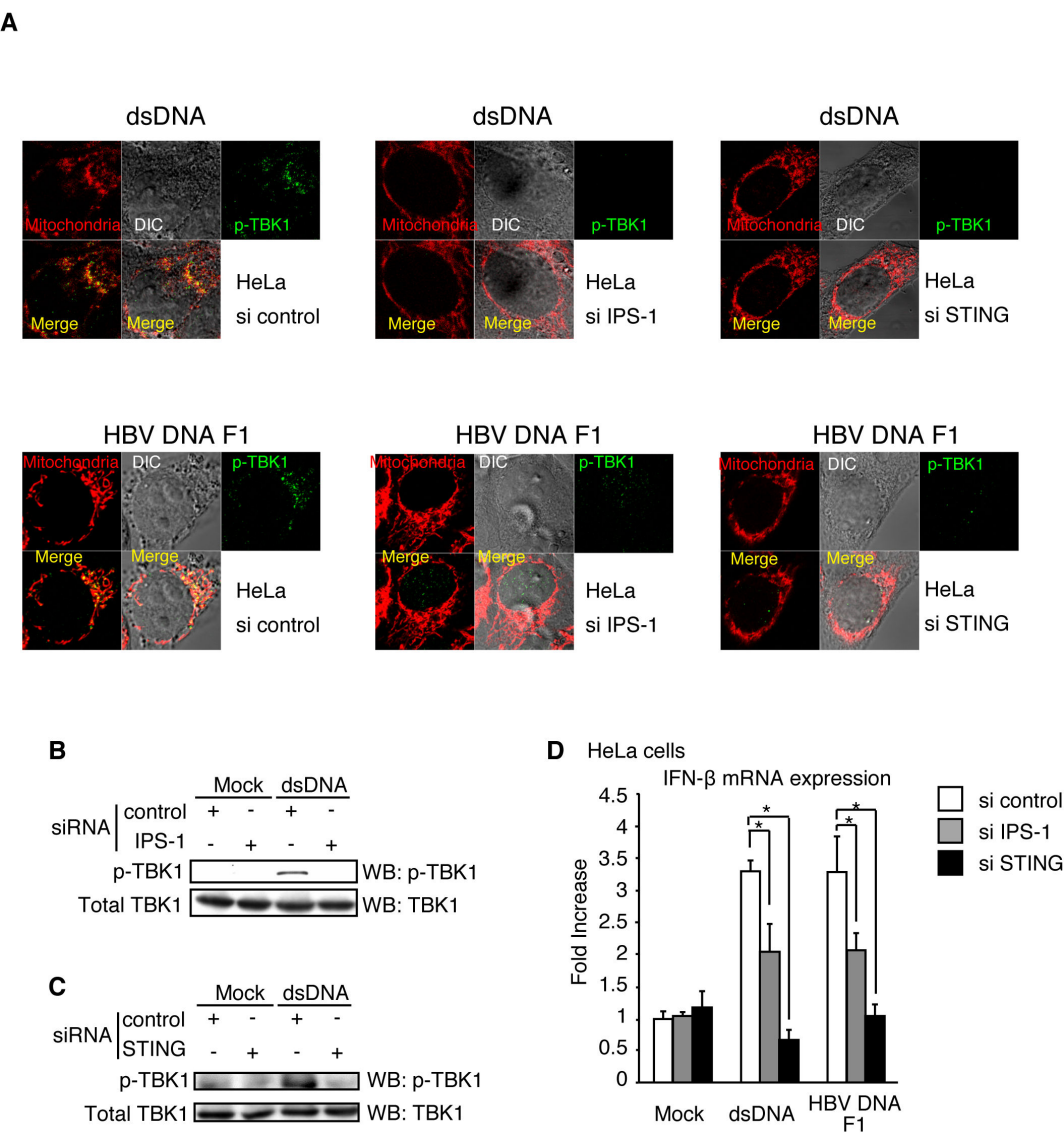
p-TBK1 levels in IPS-1 or STING knockdown cells. (A-C) siRNA for negative control, IPS-1, or STING were transfected into HeLa cells. At 48 h after transfection, cells were stimulated with HBV F1 fragment or salmon sperm DNA for 6 h. Cells were fixed and stained with Mitotracker Red and anti-p-TBK1 antibody (A), or cell lysates were prepared and subjected to SDS-PAGE and western blotting (B and C). (D) siRNA for IPS-1 or STING were transfected into HeLa cells At 48 h after transfection, cells were stimulated with mock, HBV F1 fragment, or salmon sperm DNA (dsDNA) for 6 h. The IFN-β mRNA expression was determined by RT-qPCR.

As most p-TBK1 colocalized with IPS-1 in response to cytoplasmic DNA in HeLa cells, we investigated whether IPS-1 was required for TBK1 phosphorylation in response to cytoplasmic DNA in HeLa cells. We transfected siRNA for negative control or *IPS-1* into HeLa cells. At 48 h after transfection, cells were stimulated with the HBV DNA fragment F1 or salmon sperm dsDNA by transfection. We confirmed that siRNA for *IPS-1* markedly reduced *IPS-1* mRNA levels in both mock and dsDNA stimulated cells (Figure S3). Interestingly, siRNA for *IPS-1* reduced p-TBK1 staining in HeLa cells (Figure 9A). As STING is essential for Type I IFN expression in response to cytoplasmic DNA, we investigated the requirement for STING in TBK1 phosphorylation in response to HBV DNA. Our results showed that siRNA for *STING* reduced STING mRNA levels and abrogated p-TBK1 staining (Figure 9A and Figure S3). Next, we investigated p-TBK1 levels in cell lysates by western blotting and found that knockdown of *IPS-1* reduced p-TBK1 levels induced by DNA stimulation (Figure 9B). siRNA for *STING* also reduced p-TBK1 levels (Figure 9C) 

Next, we investigated whether siRNA for *IPS-1* or *STING* reduces IFN-β mRNA expression in response to DNA stimulation. siRNA for *IPS-1* significantly reduced IFN-β mRNA expression in response to vertebrate DNA and HBV DNA F1 fragment in HeLa cells (Figure 9D), whereas siRNA for *IPS-1* failed to reduce IFN-β mRNA expression in response to vertebrate DNA in L929 cells (Figure S3B). siRNA for *STING* also reduced IFN-β mRNA expression in HeLa cells (Figure 9D). Taken together, these data indicated that IPS-1, as well as STING, are required for TBK1 phosphorylation and efficient IFN-β mRNA expression in response to cytoplasmic DNA in HeLa cells. 

**Figure 10:**
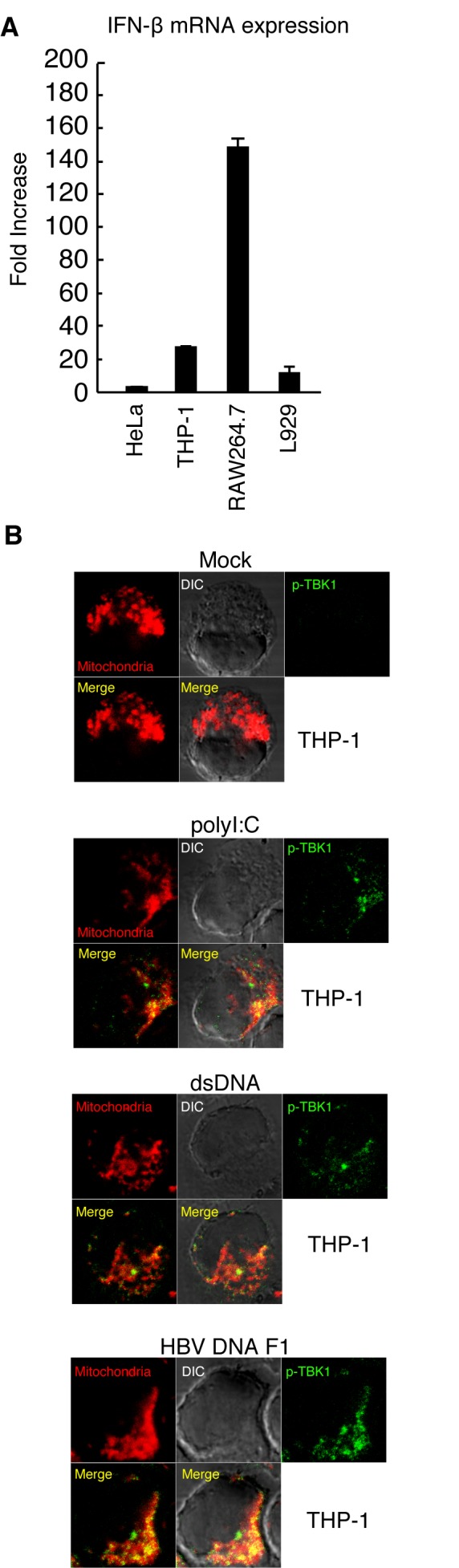
Subcellular localization of p-TBK1 in response to polyI:C or DNA in THP-1 cells. (A) HeLa, THP-1, Raw264.7, and L929 cells were stimulate with salmon sperm DNA for 6 h by transfection. The fold increase of IFN-β mRNA expression in response to DNA was determined by RT-qPCR. (B) THP-1 cells were stimulated with mock, polyI:C, salmon sperm DNA (dsDNA), or HBV DNA F1 for 3 h. Cells were fixed and stained with Mitotracker Red and anti-p-TBK1 antibody.

Next, we compared dsDNA-induced type I IFN mRNA expressions among human and mouse cells. HeLa cells were less responsive to DNA stimulation compared to THP-1, L929, and RAW264.7 cells (Figure 10A). Therefore, there is a possibility that mitochondrial localization might correlate with a lack of strong response of the cells to the cytoplasmic DNA stimulation. To test this possibility, we investigated p-TBK1 subcellular localization in THP-1, which efficiently expressed IFN-β mRNA in response to DNA stimulation as L929 cells (Figure 10A). p-TBK1 showed mitochondrial localization in response to salmon sperm DNA or HBV F1 DNA fragment in THP-1 cells (Figure 10B). This observation weakened the possibility. 

## Discussion

Autophosphorylation of TBK1 is essential for Type I IFN expression [28]. Here, we demonstrated that p-TBK1 is localized on mitochondria in response to cytoplasmic DNA in HeLa and HepG2 cells. p-TBK1 induced by DNA stimulation in HeLa and HepG2 cells co-localized with IPS-1 and MFN-1. Moreover, knockdown of *IPS-1* reduced p-TBK1 levels in response to viral DNA in HeLa cells. These data indicate that IPS-1 plays a crucial role in the phosphorylation of TBK1 in response to cytoplasmic DNA in the human cells. In contrast, p-TBK1 did not localize on mitochondria in the mouse or tree shrew cells that we tested. This is consistent with a previous knockout mouse study that showed that IPS-1 is dispensable for the response to cytoplasmic DNA [33]. Thus, there appears to be species-specific mechanisms of Type I IFN production in response to cytoplasmic DNA. However, we do not exclude the possibility that some of human primary or immortalized cells do not exhibit IPS-1 mitochondrial localization in response to cytoplasmic viral DNA. Taniguchi and colleagues firstly reported cell type-specific roles of a DNA sensor, DAI, in a cytoplasmic DNA sensing pathway [32,37]. Later, other groups reported several other DNA sensors using various types of cells [38]. Recently Chen and colleagues showed that cGAS is essential for type I IFN production in response to poly(dA:dT) in plasmacytoid DC but not in lung fibroblasts [39]. Their findings suggested that RNA polymerase III – RIG-I pathway plays a major role in sensing DNA in lung fibroblasts [24]. Thus, these observations indicate that there are several cell type-specific DNA sensing pathways. Our observations of cell type-specific p-TBK1 localization in response to cytoplasmic DNA also support this model. 

Gale and colleagues previously reported that a major site of IPS-1 signaling is MAMs [8]. TBK1 autophosphorylation in response to HBV DNA required both IPS-1 and STING in human cells that we tested. Considering that IPS-1 and STING localize on the mitochondria and ER, respectively, it is expected that TBK1 autophosphorylation occurs within MAMs where the ER associates with mitochondria in response to cytoplasmic viral DNA in HeLa and HepG2 cells. Indeed, a fraction of cellular p-TBK1 was localized on MAMs. It is likely that TBK1 moved to mitochondria after autophosphorylation in human cells. In contrast, mouse and tree shrew TBK1 appears to be phosphorylated on ER where STING localizes. The biological significance of the mitochondrial or ER localization of p-TBK1 is unclear. A previous report demonstrated that peroxisomal IPS-1 triggers rapid expression of ISGs, whereas mitochondrial IPS-1 triggers delayed ISG and IFN expression [7]. Thus, as observed with IPS-1, differential p-TBK1 placement may allow the cell to diversify signaling pathways. 

When human STING or IPS-1 were transfected into mouse cells, p-TBK1 did not localize on the mitochondria in response to cytoplasmic DNA. Thus, the differences in the STING and IPS-1 protein sequences between humans and mice are not a cause of the cell type-specific localization of p-TBK1. It is possible that the difference in TBK1 protein sequence between humans and mice determines the cell type specific localization of p-TBK1. Another possibility is that an unknown factor of human or mouse associate with TBK1 or p-TBK1. Our current knowledge cannot explain the cell type-specific localization of p-TBK1 in response to cytoplasmic DNA stimulation. Further study is required to reveal the precise mechanisms used by cytoplasmic DNA sensing pathways. 

## Materials and Methods

### Cells, viruses, and reagents

A tree shrew (*Tupaia belangeri*) fibroblast cell line, T-23 (clone 8) cells were obtained from JCRB. T-23 and HepG2 cells were cultured in Dulbecco’s modified Eagle’s medium low glucose medium (D-MEM) with 10% heat-inactivated fetal calf serum (FCS) (Invitrogen). HeLa cells were cultured in minimum Eagle’s medium with 2 mM L-glutamine and 10% heat-inactivated FCS. Protocols for the isolation and culture conditions of mouse hepatocytes have been described previously [34]. L929 and RAW264.7 cells were cultured in RPMI1640 with 10% of FCS. Salmon sperm DNA and polyI:C were purchased from Invitrogen or GE Healthcare, respectively. HSV-1 K strain was amplified using Vero cells. To determine viral titers, we performed plaque assay using Vero cells. Vero cells were cultured in D-MEM with 10% of FCS. THP-1 cells were cultured in RPMI1640 with 10 % FCS and 0.1 % 2-mercaptoethanol.

### Confocal Microscopy

Phospho-TBK1/NAK (Ser 172) (D52C2) XP rabbit mAb were purchased from Cell Signaling. Anti-Presenilin 1 Ab [APS11], anti-NAK (TBK1) Ab (EP611Y), anti-mitofusin 1 Ab, and anti-G3BP Ab were purchased from Abcam. Anti-FLAG antibody was purchased from Sigma-Aldrich. Anti-HA Ab [HA1.1] was purchased from COVANCE. Mitotracker Red was purchased from Life Technologies. Cells were fixed with 3% of formaldehyde in 1x PBS for 30 min, and permeabilized with 0.2% Triton X-100 in 1 x PBS for 15 min. In case of PSEN-1 staining, fixed cells were permeabilized with 0.5% of saponin in 1x PBS with 1 % BSA for 30 min. For blocking, 1% BSA in PBS was used for 30 min. The cells were labeled with the indicated primary antibody for 60 min at room temperature. After washing four times with 1% BSA in PBS, cells were incubated with an Alexa-conjugated secondary antibody and 1% BSA in PBS for 30 min at room temperature, and then were washed four times with 1% BSA in PBS. Samples were mounted on glass slides using Prolong Gold (Invitrogen). Cells were visualized at a magnification of ×63 with an LSM510 META microscope (Zeiss). Colocalization of coefficients and intensity histograms were determined using LSM510 ZEISS LSM Image examiner software. 

### Plasmids

RIG-I CARDs expression vector (dRIG-I) has been described previously [40]. Human *IPS-1* cDNA encoding the full-length ORF was cloned into a pEF-BOS multi-cloning site, and an HA sequence was inserted just before the STOP codon. Human *STING* cDNA that encoded a full-length ORF was cloned into a pEF-BOS multi-cloning site, and a FLAG-tag sequence was inserted just before the STOP codon. The plasmids were sequenced to confirm that there were no PCR errors. 

### HBV DNA preparation and quantitative PCR

HBV DNA fragments were obtained by PCR using HBV DNA as a template. A plasmid carrying HBV full-length genomic DNA were kindly gifted from Chayama K. Primer sequences were as follows: F1 forward, TGC AAC TTT TTC ACC TCT GC; F1 reverse, TCT CCT TTT TTC ATT AAC TG; F2 forward, TTA AAA TTA ATT ATG CCT GC; F2 reverse, AAC AAG AAA AAC CCC GCC TG; F3 forward, GAC AAG AAT CCT CAC AAT AC; F3 reverse, TGT ACA ATA TGT TCT TGT GG; F4 forward, AAA AAT CAA GCA ATG TTT TC; and F4 reverse, ATT AGG CAG AGG TGA AAA AG. Amplified DNA fragments were purified using a Gel Extraction kit (Qiagen). For quantitative PCR (qPCR), total RNA was extracted using TRIZOL reagent (Invitrogen), after which 0.1-1 μg of RNA was reverse-transcribed using a high capacity cDNA transcription kit with an RNase inhibitor kit (Applied Biosystems) according to the manufacturer’s instructions. qPCR was performed using a Step 1 real time PCR system (Applied Biosystems). The expression of cytokines mRNA was normalized to that of *GAPDH* or *β-actin* mRNA, and the fold-increase was determined by dividing the expressions in each sample by that of the wild-type at 0 h. The primers used for qPCR were described previously [40,41].

## Supporting Information

Figure S1 **HeLa cells were transfected with HA-tagged IPS-1 (**A**) or FLAG-tagged STING (**B**).** At 24 h after transfection, cells were mock-stimulated for 6 h, and then fixed and stained with anti-p-TBK1 and HA or FLAG Abs.
(TIF)

Figure S2 **HepG2 cells were transfected with a vector carrying 1.4 x HBV genomic DNA.** Total RNA was extracted at indicated hours. IFN-β mRNA expression was determined by RT-qPCR.
(TIF)

Figure S3 **siRNAs for control, IPS-1, or STING were transfected into HeLa and L929 cells.** 48 h after transfection, cells were stimulated with or without dsDNA for 6 h. Total RNA was extracted with TRIZOL, and RT-qPCR was performed. IPS-1 and STING mRNA expressions were normalized with β-actin mRNA expression. Relative ratio was calculated by dividing each ratio by the ratio of the “mock si control” sample. The target sequences of siRNA for human *IPS-1* and *STING* are: CCA AAG UGC CUA CCA CCU U and GGA UUC GAA CUU ACA AUC A, respectively. The target sequence of siRNA for mouse *IPS-1* and *STING* are: UGU　UGC　CUC　UGU　UCC　CAUA and GCA CAU UCG UCA GGA AGA A, respectively. Silencer Select siRNAs were purchased from Life Technologies.
(TIF)

## References

[1] Loo YM, Gale M Jr. (2011) Immune signaling by RIG-I-like receptors. Immunity 34: 680-692. doi:10.1016/j.immuni.2011.05.003. PubMed: 21616437. 21616437 PMC3177755

[2] Onomoto K, Jogi M, Yoo JS, Narita R, Morimoto S et al. (2012) Critical role of an antiviral stress granule containing RIG-I and PKR in viral detection and innate immunity. PLOS ONE 7: e43031. doi:10.1371/journal.pone.0043031. PubMed: 22912779. 22912779 PMC3418241

[3] Xu LG, Wang YY, Han KJ, Li LY, Zhai Z et al. (2005) VISA is an adapter protein required for virus-triggered IFN-beta signaling. Mol Cell 19: 727-740. doi:10.1016/j.molcel.2005.08.014. PubMed: 16153868. 16153868

[4] Seth RB, Sun L, Ea CK, Chen ZJ (2005) Identification and characterization of MAVS, a mitochondrial antiviral signaling protein that activates NF-kappaB and IRF 3. Cell 122: 669-682. doi:10.1016/j.cell.2005.08.012. PubMed: 16125763. 16125763

[5] Meylan E, Curran J, Hofmann K, Moradpour D, Binder M et al. (2005) Cardif is an adaptor protein in the RIG-I antiviral pathway and is targeted by hepatitis C virus. Nature 437: 1167-1172. doi:10.1038/nature04193. PubMed: 16177806. 16177806

[6] Kawai T, Takahashi K, Sato S, Coban C, Kumar H et al. (2005) IPS-1, an adaptor triggering RIG-I- and Mda5-mediated type I interferon induction. Nat Immunol 6: 981-988. doi:10.1038/ni1243. PubMed: 16127453. 16127453

[7] Dixit E, Boulant S, Zhang Y, Lee AS, Odendall C et al. (2010) Peroxisomes are signaling platforms for antiviral innate immunity. Cell 141: 668-681. doi:10.1016/j.cell.2010.04.018. PubMed: 20451243. 20451243 PMC3670185

[8] Horner SM, Liu HM, Park HS, Briley J, Gale M Jr. (2011) Mitochondrial-associated endoplasmic reticulum membranes (MAM) form innate immune synapses and are targeted by hepatitis C virus. Proc Natl Acad Sci U S A 108: 14590-14595. doi:10.1073/pnas.1110133108. PubMed: 21844353. 21844353 PMC3167523

[9] Onoguchi K, Onomoto K, Takamatsu S, Jogi M, Takemura A et al. (2010) Virus-infection or 5'ppp-RNA activates antiviral signal through redistribution of IPS-1 mediated by MFN1. PLoS Pathog 6: e1001012 PubMed: 20661427. 20661427 10.1371/journal.ppat.1001012 PMC2908619

[10] Yasukawa K, Oshiumi H, Takeda M, Ishihara N, Yanagi Y et al. (2009) Mitofusin 2 inhibits mitochondrial antiviral signaling. Sci Signal 2: ra47 PubMed: 19690333. 19690333 10.1126/scisignal.2000287

[11] Hou F, Sun L, Zheng H, Skaug B, Jiang QX et al. (2011) MAVS forms functional prion-like aggregates to activate and propagate antiviral innate immune response. Cell 146: 448-461. doi:10.1016/j.cell.2011.06.041. PubMed: 21782231. 21782231 PMC3179916

[12] Perry AK, Chow EK, Goodnough JB, Yeh WC, Cheng G (2004) Differential requirement for TANK-binding kinase-1 in type I interferon responses to toll-like receptor activation and viral infection. J Exp Med 199: 1651-1658. doi:10.1084/jem.20040528. PubMed: 15210743. 15210743 PMC2212814

[13] Hemmi H, Takeuchi O, Sato S, Yamamoto M, Kaisho T et al. (2004) The roles of two IkappaB kinase-related kinases in lipopolysaccharide and double stranded RNA signaling and viral infection. J Exp Med 199: 1641-1650. doi:10.1084/jem.20040520. PubMed: 15210742. 15210742 PMC2212809

[14] Oshiumi H, Matsumoto M, Funami K, Akazawa T, Seya T (2003) TICAM-1, an adaptor molecule that participates in Toll-like receptor 3-mediated interferon-beta induction. Nat Immunol 4: 161-167. doi:10.1038/ni886. PubMed: 12539043. 12539043

[15] Matsumoto M, Funami K, Tanabe M, Oshiumi H, Shingai M et al. (2003) Subcellular localization of Toll-like receptor 3 in human dendritic cells. J Immunol 171: 3154-3162. PubMed: 12960343. 12960343 10.4049/jimmunol.171.6.3154

[16] Alexopoulou L, Holt AC, Medzhitov R, Flavell RA (2001) Recognition of double-stranded RNA and activation of NF-kappaB by Toll-like receptor 3. Nature 413: 732-738. doi:10.1038/35099560. PubMed: 11607032. 11607032

[17] Sun L, Wu J, Du F, Chen X, Chen ZJ (2013) Cyclic GMP-AMP synthase is a cytosolic DNA sensor that activates the type I interferon pathway. Science 339: 786-791. doi:10.1126/science.1232458. PubMed: 23258413. 23258413 PMC3863629

[18] Kondo T, Kobayashi J, Saitoh T, Maruyama K, Ishii KJ et al. (2013) DNA damage sensor MRE11 recognizes cytosolic double-stranded DNA and induces type I interferon by regulating STING trafficking. Proc Natl Acad Sci U S A 110: 2969-2974. doi:10.1073/pnas.1222694110. PubMed: 23388631. 23388631 PMC3581880

[19] Desmet CJ, Ishii KJ (2012) Nucleic acid sensing at the interface between innate and adaptive immunity in vaccination. Nat Rev Immunol 12: 479-491. doi:10.1038/nri3247. PubMed: 22728526. 22728526

[20] Stetson DB, Medzhitov R (2006) Recognition of cytosolic DNA activates an IRF3-dependent innate immune response. Immunity 24: 93-103. doi:10.1016/j.immuni.2005.12.003. PubMed: 16413926. 16413926

[21] Ishii KJ, Coban C, Kato H, Takahashi K, Torii Y et al. (2006) A Toll-like receptor-independent antiviral response induced by double-stranded B-form. DNA - Nat Immunol 7: 40-48. doi:10.1038/ni1282. 16286919

[22] Choi MK, Wang Z, Ban T, Yanai H, Lu Y et al. (2009) A selective contribution of the RIG-I-like receptor pathway to type I interferon responses activated by cytosolic. DNA - Proc Natl Acad Sci U S A 106: 17870-17875. doi:10.1073/pnas.0909545106. 19805092 PMC2764914

[23] Cheng G, Zhong J, Chung J, Chisari FV (2007) Double-stranded DNA and double-stranded RNA induce a common antiviral signaling pathway in human cells. Proc Natl Acad Sci U S A 104: 9035-9040. doi:10.1073/pnas.0703285104. PubMed: 17517627. 17517627 PMC1885623

[24] Chiu YH, Macmillan JB, Chen ZJ (2009) RNA polymerase III detects cytosolic DNA and induces type I interferons through the RIG-I pathway. Cell 138: 576-591. doi:10.1016/j.cell.2009.06.015. PubMed: 19631370. 19631370 PMC2747301

[25] Ishikawa H, Ma Z, Barber GN (2009) STING regulates intracellular DNA-mediated, type I interferon-dependent innate immunity. Nature 461: 788-792. doi:10.1038/nature08476. PubMed: 19776740. 19776740 PMC4664154

[26] Ishikawa H, Barber GN (2008) STING is an endoplasmic reticulum adaptor that facilitates innate immune signalling. Nature 455: 674-678. doi:10.1038/nature07317. PubMed: 18724357. 18724357 PMC2804933

[27] Ishii KJ, Kawagoe T, Koyama S, Matsui K, Kumar H et al. (2008) TANK-binding kinase-1 delineates innate and adaptive immune responses to DNA vaccines. Nature 451: 725-729. doi:10.1038/nature06537. PubMed: 18256672. 18256672

[28] Soulat D, Bürckstümmer T, Westermayer S, Goncalves A, Bauch A et al. (2008) The DEAD-box helicase DDX3X is a critical component of the TANK-binding kinase 1-dependent innate immune response. EMBO J 27: 2135-2146. doi:10.1038/emboj.2008.126. PubMed: 18583960. 18583960 PMC2453059

[29] Fitzgerald KA, McWhirter SM, Faia KL, Rowe DC, Latz E et al. (2003) IKKepsilon and TBK1 are essential components of the IRF3 signaling pathway. Nat Immunol 4: 491-496. doi:10.1038/ni921. PubMed: 12692549. 12692549

[30] Oshiumi H, Miyashita M, Matsumoto M, Seya T (2013) A Distinct Role of Riplet-Mediated K63-Linked Polyubiquitination of the RIG-I Repressor Domain in Human Antiviral Innate Immune Responses. PLOS Pathog 9: e1003533. 23950712 10.1371/journal.ppat.1003533 PMC3738492

[31] Yoneyama M, Kikuchi M, Natsukawa T, Shinobu N, Imaizumi T et al. (2004) The RNA helicase RIG-I has an essential function in double-stranded RNA-induced innate antiviral responses. Nat Immunol 5: 730-737. doi:10.1038/ni1087. PubMed: 15208624. 15208624

[32] Takaoka A, Wang Z, Choi MK, Yanai H, Negishi H et al. (2007) DAI (DLM-1/ZBP1) is a cytosolic DNA sensor and an activator of innate immune response. Nature 448: 501-505. doi:10.1038/nature06013. PubMed: 17618271. 17618271

[33] Kumar H, Kawai T, Kato H, Sato S, Takahashi K et al. (2006) Essential role of IPS-1 in innate immune responses against RNA viruses. J Exp Med 203: 1795-1803. doi:10.1084/jem.20060792. PubMed: 16785313. 16785313 PMC2118350

[34] Aly HH, Oshiumi H, Shime H, Matsumoto M, Wakita T et al. (2011) Development of mouse hepatocyte lines permissive for hepatitis C virus (HCV). PLOS ONE 6: e21284. doi:10.1371/journal.pone.0021284. PubMed: 21731692. 21731692 PMC3120852

[35] Taketomi M, Nishi Y, Ohkawa Y, Inui N (1986) Establishment of lung fibroblastic cell lines from a non-human primate Tupaia belangeri and their use in a forward gene mutation assay at the hypoxanthine-guanine phosphoribosyl transferase locus. Mutagenesis 1: 359-365. doi:10.1093/mutage/1.5.359. PubMed: 3331674. 3331674

[36] Kumar M, Jung SY, Hodgson AJ, Madden CR, Qin J et al. (2011) Hepatitis B virus regulatory HBx protein binds to adaptor protein IPS-1 and inhibits the activation of beta interferon. J Virol 85: 987-995. doi:10.1128/JVI.01825-10. PubMed: 21068253. 21068253 PMC3020017

[37] Wang Z, Choi MK, Ban T, Yanai H, Negishi H et al. (2008) Regulation of innate immune responses by DAI (DLM-1/ZBP1) and other DNA-sensing molecules. Proc Natl Acad Sci U S A 105: 5477-5482. doi:10.1073/pnas.0801295105. PubMed: 18375758. 18375758 PMC2291118

[38] Paludan SR, Bowie AG (2013) Immune sensing of DNA. Immunity 38: 870-880. doi:10.1016/j.immuni.2013.05.004. PubMed: 23706668. 23706668 PMC3683625

[39] Li XD, Wu J, Gao D, Wang H, Sun L et al. (2013) Pivotal roles of cGAS-cGAMP signaling in antiviral defense and immune adjuvant effects. Science 341: 1390-1394. doi:10.1126/science.1244040. PubMed: 23989956. 23989956 PMC3863637

[40] Oshiumi H, Matsumoto M, Hatakeyama S, Seya T (2009) Riplet/RNF135, a RING finger protein, ubiquitinates RIG-I to promote interferon-beta induction during the early phase of viral infection. J Biol Chem 284: 807-817. PubMed: 19017631. 19017631 10.1074/jbc.M804259200

[41] Oshiumi H, Miyashita M, Inoue N, Okabe M, Matsumoto M et al. (2010) The ubiquitin ligase Riplet is essential for RIG-I-dependent innate immune responses to RNA virus infection. Cell Host Microbe 8: 496-509. doi:10.1016/j.chom.2010.11.008. PubMed: 21147464. 21147464

