# Supplementary figures and images for: Cell Type-Specific Subcellular Localization of Phospho-TBK1 in Response to Cytoplasmic Viral DNA
Source: PLoS One. 2013 Dec 9;8(12):e83639. doi: 10.1371/journal.pone.0083639 (PMC3857317; doi:10.1371/journal.pone.0083639)

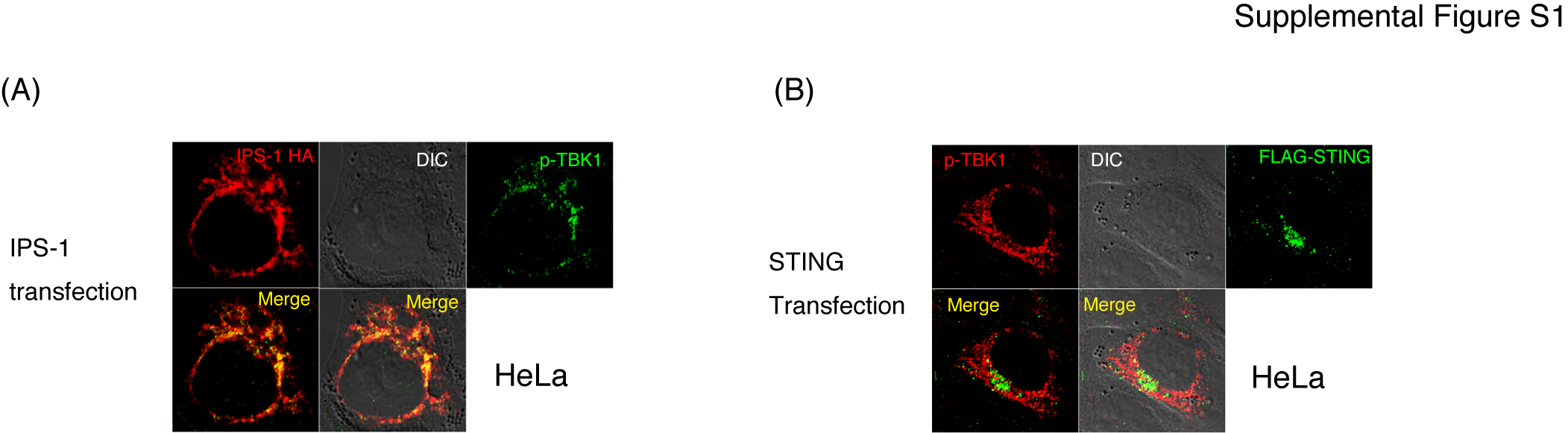

Supplement: Figure S1 — HeLa cells were transfected with HA-tagged IPS-1 (A) or FLAG-tagged STING (B). At 24 h after transfection, cells were mock-stimulated for 6 h, and then fixed and stained with anti-p-TBK1 and HA or FLAG Abs. (TIF) [file pone.0083639.s001.tif]

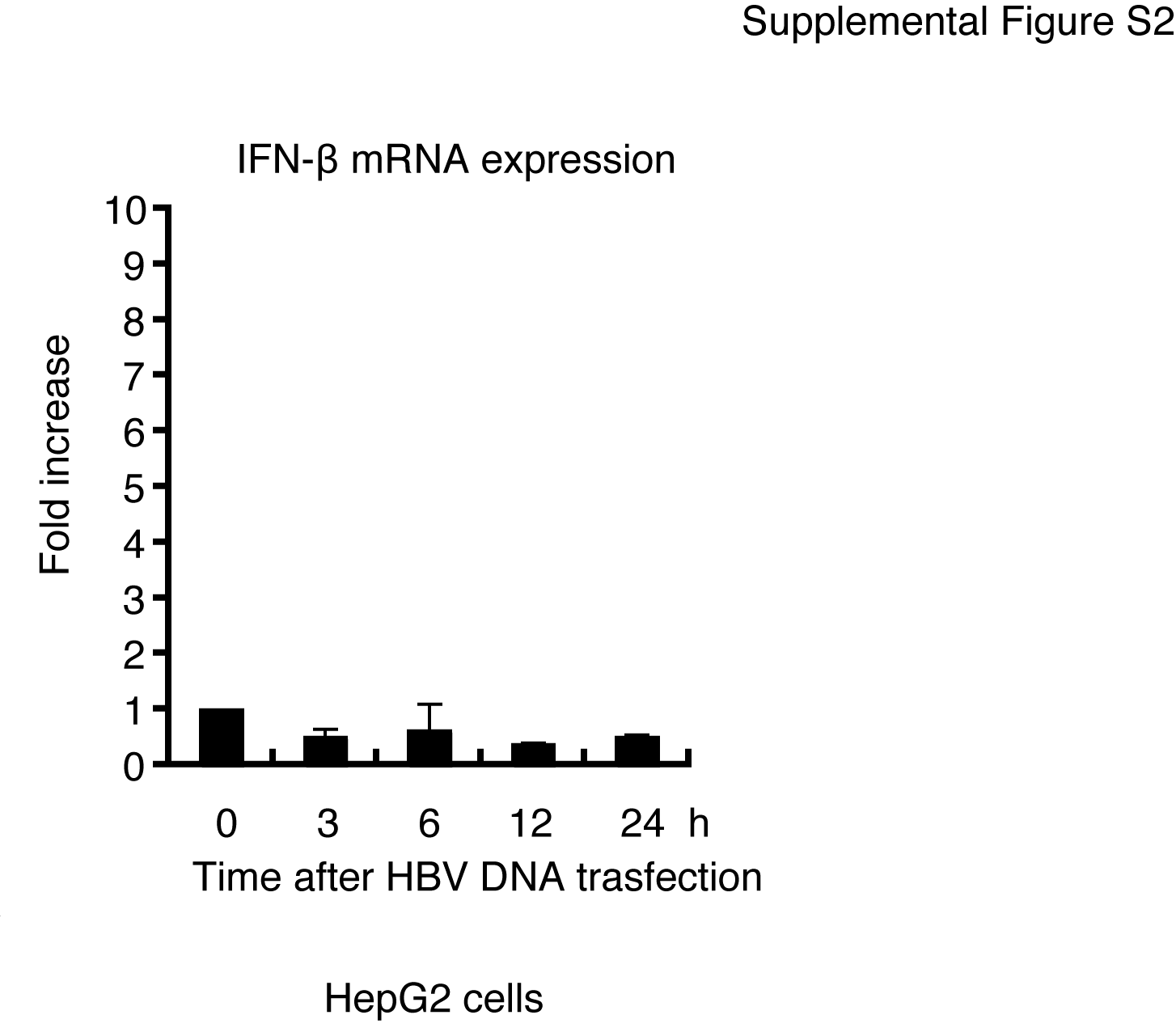

Supplement: Figure S2 — HepG2 cells were transfected with a vector carrying 1.4 x HBV genomic DNA. Total RNA was extracted at indicated hours. IFN-β mRNA expression was determined by RT-qPCR. (TIF) [file pone.0083639.s002.tif]

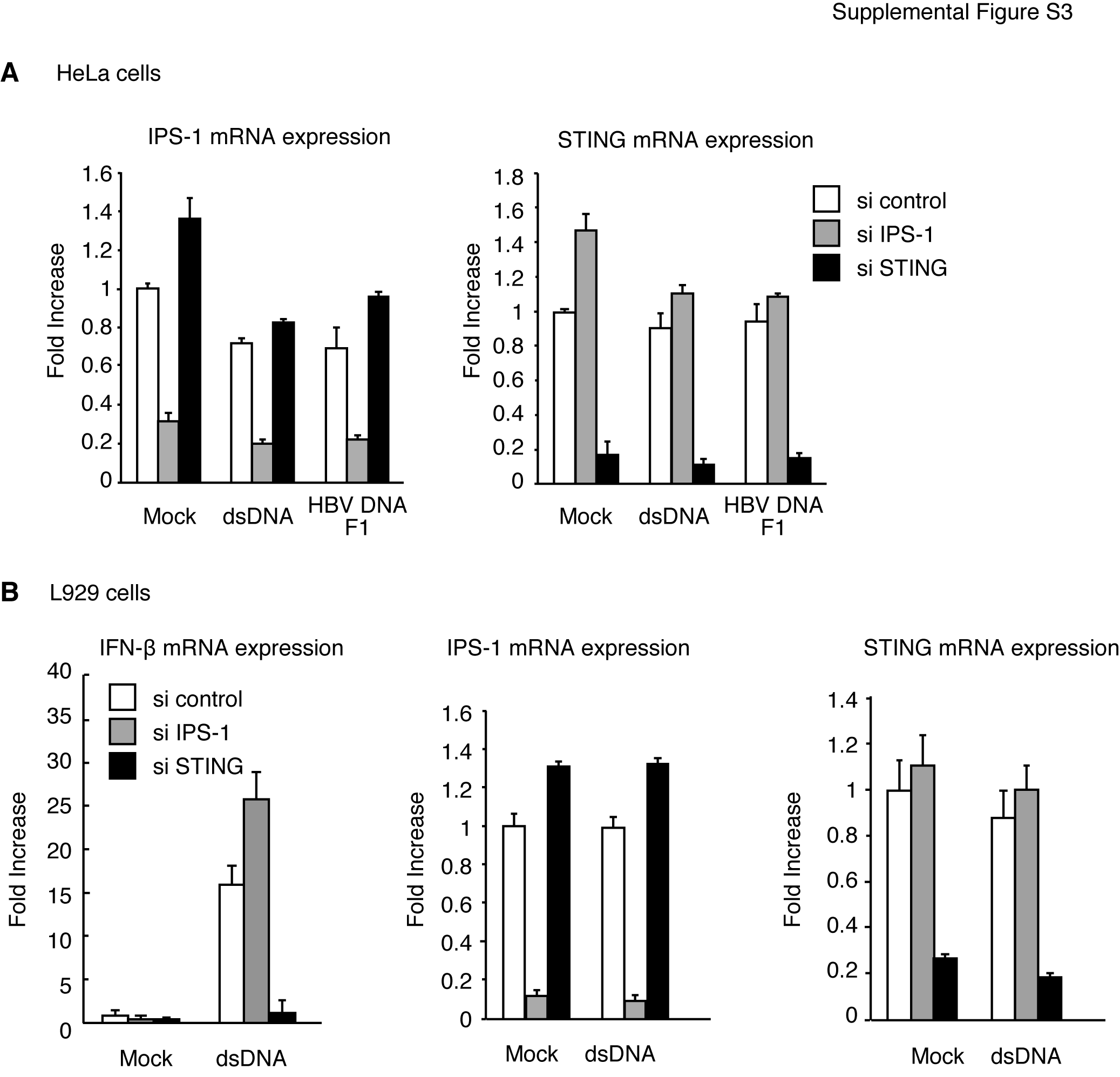

Supplement: Figure S3 — siRNAs for control, IPS-1, or STING were transfected into HeLa and L929 cells. 48 h after transfection, cells were stimulated with or without dsDNA for 6 h. Total RNA was extracted with TRIZOL, and RT-qPCR was performed. IPS-1 and STING mRNA expressions were normalized with β-actin mRNA expression. Relative ratio was calculated by dividing each ratio by the ratio of the “mock si control” sample. The target sequences of siRNA for human IPS-1 and STING are: CCA AAG UGC CUA CCA CCU U and GGA UUC GAA CUU ACA AUC A, respectively. The target sequence of siRNA for mouse IPS-1 and STING are: UGU UGC CUC UGU UCC CAUA and GCA CAU UCG UCA GGA AGA A, respectively. Silencer Select siRNAs were purchased from Life Technologies. (TIF) [file pone.0083639.s003.tif]
